# Population structure, genetic diversity and prolificacy in pishan red sheep under an extreme desert environment

**DOI:** 10.3389/fgene.2023.1092066

**Published:** 2023-04-11

**Authors:** Cheng-long Zhang, Jihu Zhang, Mirenisa Tuersuntuoheti, Qianqian Chang, Shudong Liu

**Affiliations:** ^1^ College of Animal Science and Technology, Tarim University, Alar, China; ^2^ Key Laboratory of Tarim Animal Husbandry Science and Technology, Xinjiang Production and Construction Corps, Alar, China

**Keywords:** desert environment, genomic selection, linkage disequilibrium, perennial estrus, litter size

## Abstract

Extreme environmental conditions are a major challenge for livestock production. Changes in climate conditions, especially those that lead to extreme weather, can reduce livestock production. The screening of genes and molecular markers is of great significance to explore the genetic mechanism of sheep prolificacy traits in Taklimakan Desert environment. We selected healthy adult Pishan Red Sheep (PRS) and Qira Black Sheep (QR) which live in Taklimakan Desert environment, collected blood from jugular vein, extracted DNA, and prepared Illumina Ovine SNP50 chip. For PRS, linkage disequilibrium (LD) was calculated using the ovine SNP50 Beadchip and the effective population size (*Ne*) was estimated using SMC++. The genetic characteristics of PRS were analyzed by integrated haplotype score (iHS) and fixation index (*F*_*ST*_). The result showed that *r* ^2^ of PRS was 0.233 ± 0.280 in the range of 0–10 Kb and decreased with increasing distances. SMC++ tested that the *Ne* of PRS remained at 236.99 in recent generations. 184 genes were screened out under iHS 1% threshold, and 1148 genes were screened out with *F*_*ST*_ under the 5% threshold, and 29 genes were obtained from the intersection of the two gene sets. In this study, the genetic characteristics of PRS and QR were compared by ovine genome chip, and the related excellent genes were searched, providing reference for the protection of sheep germplasm resources and molecular breeding in a desert environment.

## 1 Introduction

Sheep is one of the earliest domesticated animals in the world, and also one of the most successful animals domesticated by human beings in the Neolithic age. After long-term domestication and different environments, sheep have great changes in morphology, physiology and behavior. Pishan Red sheep (PRS) living in the Taklimakan Desert is characterized by perennial estrus and multiple fetuses after long-term selection by nature and people. In addition to its high-quality production traits, PRS is also a rare breed of sheep because its origin is on the southern edge of the Taklimakan Desert and north of the Karakoram Mountains (Lv et al., 2020). Pishan red sheep is a local sheep breed formed under the local cultural and geographical conditions. The origin and formation history of PRS is not fully understood. Therefore, to dig the genetic structure and molecular markers of PRS can better protect PRS.

Linkage disequilibrium (LD) can improve the accuracy of genomic association analysis and predict marker regions (Liu et al., 2017). LD decay patterns also provide information about the evolutionary history of the population and can be used to estimate ancestral effective population size (*Ne*) (Tenesa et al., 2007). *Ne* and other genetic events can also influence the extent of LD in the population (Wang, 2005). Therefore, LD helps in understanding the selection patterns experienced by individual breeds. Currently, LD estimates have been reported in several studies for a variety of livestock species, such as cattle (Porto-Neto et al., 2014), pigs (Uimari and Tapio, 2011), horses (Corbin et al., 2010), chickens (Rao et al., 2008) and sheep (Kijas et al., 2014).

The selected regions were searched through different chromosomes to provide a molecular genetic basis for sheep protection. Compared with traditional selection methods, genomics can be evaluated early with higher accuracy (Hayes et al., 2013). Voight and Kudaravalli proposed an integrated haplotype score (iHS) test based on extended haplotype homozygosity (Voight et al., 2006). The incomparability of test statistics caused by differences in recombination rates between different chromosome segments was corrected by calculating the Extended Haplotype Homozygosity (EHH) statistics and genetic distance integration. Fixation index (*F*_*ST*_) is used to measure the degree of population differentiation, indicating that there are obvious allele frequency differences between populations (Akey et al., 2002). Genetic drift and selection process can usually cause the genetic differentiation between populations. This method is suitable for selection signal detection of multiple populations. Now, it is necessary to selectively intervene in the breeding work of PRS through genomic selection technology to guarantee a better inheritance of the excellent production traits of PRS to future generations.

In order to analyze the genetic mechanism of prolific traits in PRS under desert environment, we selected prolific PRS and singleton pregnancy Qira Black Sheep (QR) under relevant survival background as research objects. Genetic basis of PRS was analyzed based on LD and *Ne* and the genetic mechanism of prolific traits in Taklimakan Desert was explored by using genomic selection method.

## 2 Materials and methods

### 2.1 Animal care

This work was conducted in accordance with the specifications of the Ethics Committee of Tarim University of Science and Technology (SYXK 2020-009).

### 2.2 Animal collection

33 PRS (polyembryony) and 40 QR (singleton pregnancy) were randomly selected from Pishan County and Cele County in Hetian region and all of them were healthy adult sheep with no genetic relationship. Blood samples were collected from the jugular vein and DNA was extracted with a DNA kit (Tiangen Biotech Co. Ltd., Beijing, China). The samples were sent to Beijing Compass Agritechnology Co., Ltd. to prepare the Illumina Ovine SNP50 Beadchip (The number of SNPs in this chip is 50 k). The validation samples were 130 PRS (first lambing) from Pishan Farm, with the same feeding conditions, 1.5–2.5 years old, no relationship.

### 2.3 Genotyping and data quality control

Genome Studio software was used to process the preliminary data results and obtain the VCF files. Plink (Purcell et al., 2007) software was used for quality control. Unqualified SNP sites were eliminated. The quality control criteria of this study were as follows: 1) individual detection rate >0.95, 2) SNP detection rate >0.95, and 3) Hardy-Weinberg equilibrium (HWE) with *p* values $\geq 10^{-6}$ .

### 2.4 LD calculation method

LD is the basis of association analysis, and the analysis of LD between loci helps to understand the LD level of the PRS genome. Since *r* ^2^ is more capable of objectively reflecting the LD between different loci, it was adopted in this study as the LD measurement standard. The LD values ranged from 0 to 1. As the LD level increased with the value of *r* ^2^, the linkage degree increased. The calculation formula of *r* ^2^ is as follows (Hill, 1974):

$$r^{2}=\frac{{\left(P_{A1B1}-P_{A1}\cdot P_{B1}\right)}^{2}}{P_{A1}\cdot \left(1-P_{A1}\right)\cdot P_{B1}\cdot \left(1-P_{B1}\right)}$$

Where PA1 and PB1 are the frequency of the first allele at the two marker loci and the haplotype frequency formed between alleles. The correlation coefficient (*r* ^2^ mean) of alleles was calculated to measure the level of linkage disequilibrium (LD) using PopLDdecay V3.41 (Zhang et al., 2018), and perl scripts were used to visualize the results.

### 2.5 Estimation of effective population size

The SMC++ (Terhorst et al., 2016) method was used to estimate *Ne*. The population size history and splitting time of the PRS can be predicted with SMC++. A new spline regularization scheme was adopted in this method, significantly reducing estimation errors. The conversion of each VCF file into an input file in SMC++ format was made using the vcf2smc script distributed by SMC++. All the simulations were performed under the initial condition of a mutation rate of 1.25 × 10^−8^.

### 2.6 Genetic diversity and population structure

The genotypic data after quality control was subjected to Principal Component Analysis (PCA) using MingPCACluster (https://github.com/hewm2008/MingPCACluster). The VCF2Dis v1.09 (https://github.com/BGI-shenzhen/VCF2Dis) was used to calculate the P distance matrix, and then the NJ-tree was constructed by ATGC:FastME (http://www.atgc-montpellier.fr/fastme) program. Genetic admixture calculations were performed using Admixture (Patterson et al., 2012).

### 2.7 Fixation index

*F*_*ST*_ is used to measure the degree of population differentiation and can reflect the level of species population differentiation. This method is suitable for selective signal detection of multiple populations and as follows:

$$F_{ST}=\frac{MSP-MSG}{MSP+\left(nc-1\right)MSG}$$

Where, MSG is the mean square of error within the population, MSP is the mean square of error between the populations, and nc is the average sample size between the populations after correction. By using a sliding window with a window size of 50 Kb and a sliding step size of 25 Kb, the *F*_*ST*_ value of each sliding window SNP is calculated. Vcftools was used to calculate the *F*_*ST*_ value for each window, and then CMplot was used to plot Manhattan Yin et al. (2021).

### 2.8 Integrated haplotype score

An intrapopulation selective genomic sweep analysis was performed on all individuals using iHS. iHS is an alternative EHH statistic using a single marker loci haploid type. It is defined as the core in the site, the expansion of the ancestors in the core loci alleles in the haploid type, and new mutant alleles in the extension of the haploid type EHH statistics for the integral genetic distance. It is possible to calculate the ratio between the previously mentioned genetic metrics to select a signal detection statistic using this method, as expressed by Voight et al. (2006):

$$IHS=\frac{unIHS-mean\left(unIHS|ps\right)}{sd\left(unIHS|ps\right)}$$

uniHS is:

$$unIHS=In\left(\frac{IHH_{A}}{IHH_{D}}\right)$$

Where IHH (Integrated EHH) refers to integrating genetic distance with EHH; A is the ancestral allele, and D is the newly derived allele.

### 2.9 Enrichment analysis of candidate genes

The iHS results and *F*_*ST*_ results were selected for intersection analysis, with annotations with the sheep genome Ovis Oar_v4.0. Gene functional annotation was performed referencing the NCBI databases (http://www.ncbi.nlm.nih.gov/gene) and OMIM database (http://www.ncbi.nlm.nih.gov/omim). The g:Profiler (https://biit.cs.ut.ee/gprofiler/gost) was used for autosomal enrichment of candidate genes for GO and Reactome/KEGG pathway analysis.

### 2.10 PCR amplification of BMPR1B

The *Fec* ^*B*^ locus of the BMPR1B gene in 130 PRS was amplified by PCR according to the primers shown in Supplementary Material. After the PCR products were detected by 1.5% agarose gel electrophoresis, all qualified PCR products were sent to Beijing Compass Agritechnology Co., Ltd. for DNA sequencing and genotype identification.

## 3 Results

### 3.1 Descriptive statistics

Genotypic quality control was conducted on the SNPs of the 33 PRS and 40 QR used in the experiment. After the unqualified SNPs were removed, there were 49,219 informative SNPs in the PRS and QR population.

### 3.2 The extent of genome-wide LD and effective population size of PRS

**FIGURE 1:**
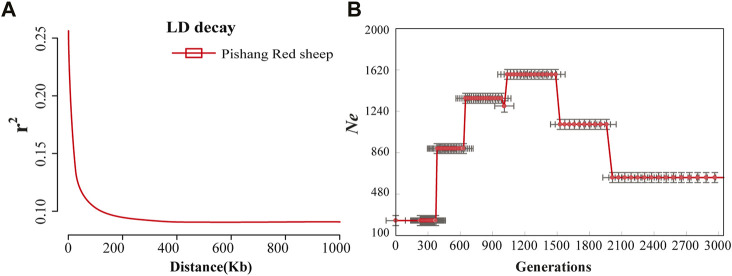
LD decay and *Ne* in PRS. **(A)**: Distribution of average *r* ^2^ values for PRS with respect to physical distance (kb) in 1,000 kb windows. **(B)**: Genome-wide LD (*r* ^2^) between SNPs was used to estimate the *Ne* of the PRS.

When LD was calculated, the distance between markers was set in the 0–1 Mb (0–10, 10–25, 25–50, 50–100, 100–500 Kb, 0.5–1 Mb) autosomal range (Table 1). The average *r* ^2^ decreased with increasing physical distance (Figure 1A). When the distance between markers was 10–25 Kb, the average *r* ^2^ value was 0.151 ± 0.200. Compared with the average *r* ^2^ value between SNPs within 0–10 Kb (0.233 ± 0.280), the difference was 0.082, much larger than the average *r* ^2^ value between other adjacent distance regions. When the distance increased to 50–100 Kb, the *r* ^2^ value was lower than 0.1, indicating that the LD was weak.

**TABLE 1:** LD statistical analysis between different distances (0–1 Mb).

Distance	Average *r* ^2^	Number of SNP pairs	Proportion of *r* ^2^ > 0.2	Proportion of *r* ^2^ > 0.3
0-10 Kb	0.233 ± 0.280	8561	0.364	0.288
10-25 Kb	0.151 ± 0.200	10,948	0.233	0.150
25-50 Kb	0.120 ± 0.158	18,123	0.177	0.099
50-100 Kb	0.096 ± 0.115	36,123	0.122	0.050
100-500 Kb	0.078 ± 0.080	185,353	0.075	0.019
0.5‐1 Mb	0.074 ± 0.073	368,857	0.062	0.014

*r* ^2^: denotes the extent of LD.

Based on LD, six generations *Ne* of PRS were estimated (Figure 1B). The PRS remained relatively stable at about 631.97 during the 2,500–2,000 generations. It rose to 1,119.99 with 1,500 generations, 1,328.81 with 1,000 generations, and fell to 898.12 with 500 generations. It declined even faster until recent generations stood at 236.99. During the first 200-100 years, PRS populations remained relatively stable. It was only in the last 10 years that the population increased slightly due to the introduction of measures to protect the endemic species.

### 3.3 Genetic diversity and population structure

**FIGURE 2:**
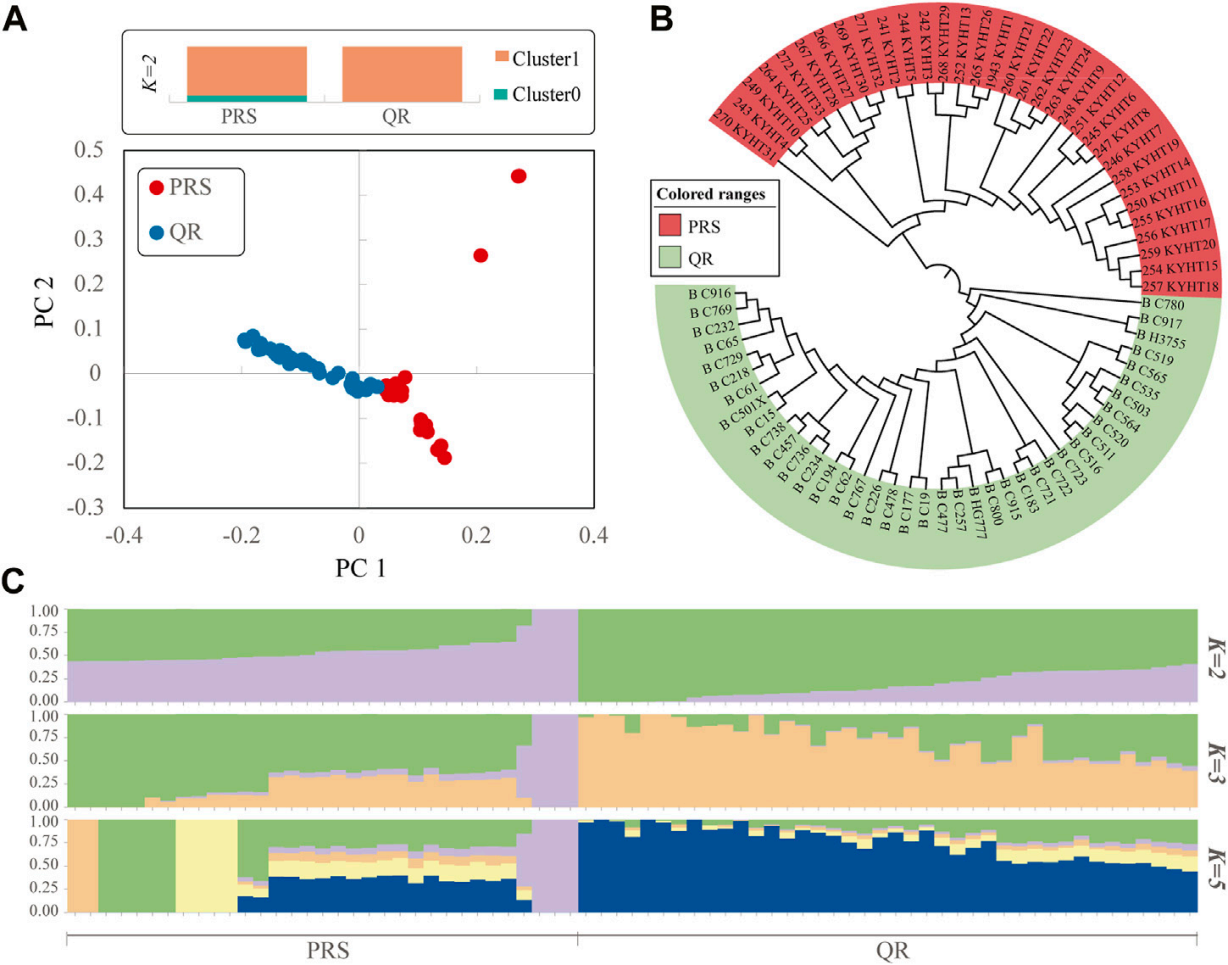
Analysis of the population structure in PRS and QR. **(A)**, Principal component analysis, k-means clustering was carried out according to the predefined subsets. **(B)**, Neighbor-joining tree. **(C)**, Analysis of Admixture with the assumed number (K = 2, 3 and 5).

PCA analysis can separate PRS from QR, and part of PRS extended outward (Figure 2A). In Figure 2A, k-means clustering was carried out according to the predefined subsets. When k = 2, the distinction was obvious. Neighbor-Joining (N-J) Tree showed that PRS and QR were divided into two varieties (Figure 2B), which was consistent with PCA analysis. Admixture analysis showed that PRS and QR had similar genetic backgrounds (Figure 2C).

### 3.4 Selective gene sweep

**FIGURE 3:**
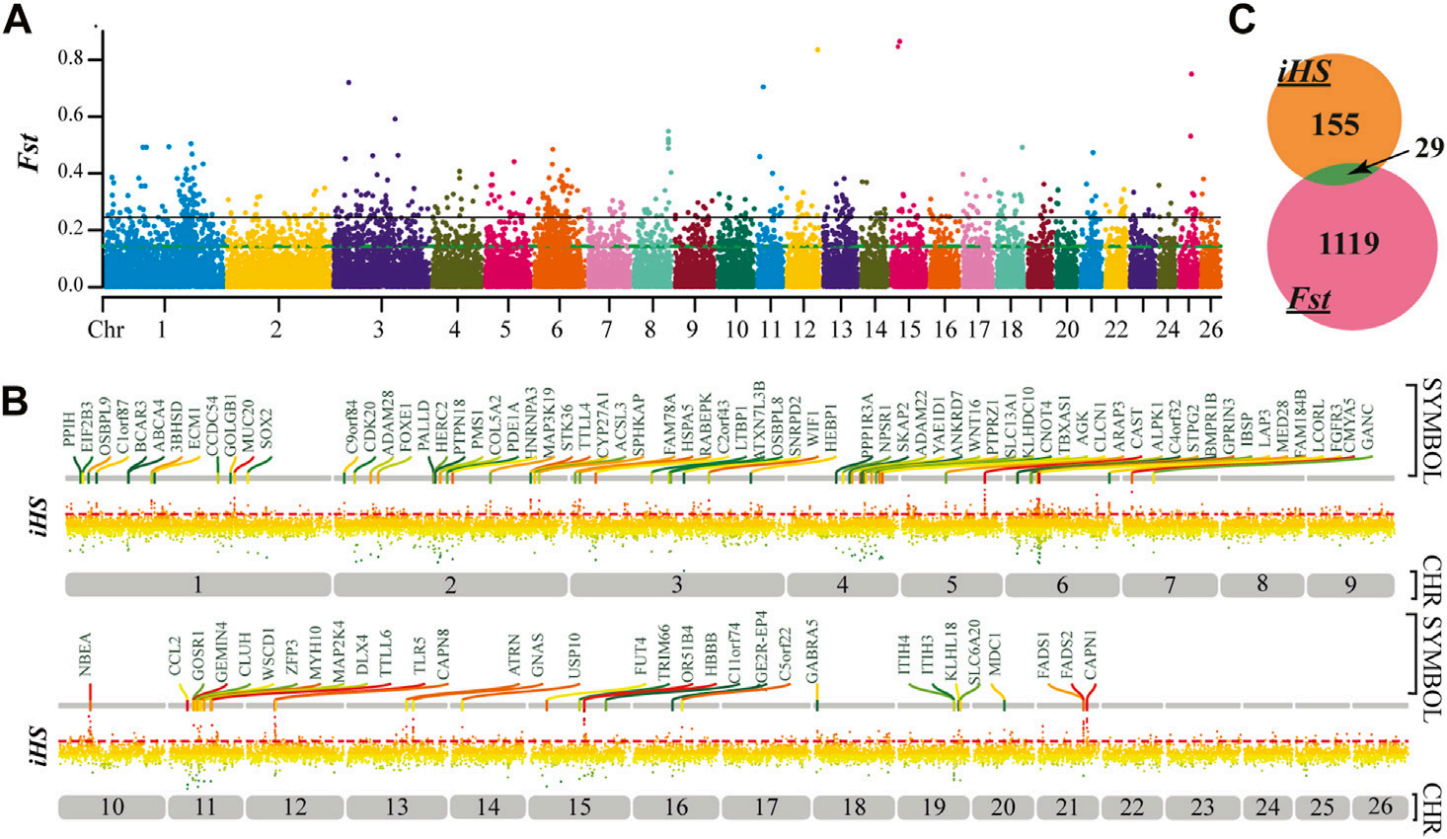
Selective gene sweep. **(A)**, *F*_*ST*_ detection results of PRS and QR. The black line represents the 1% threshold line and the green line represents the 5% threshold line. **(B)**, The 100 high-rank genes under the iHS 1% threshold. **(C)**, The intersection of *F*_*ST*_ and IHS results.

The *F*_*ST*_ values of PRS and QR were calculated, and the *F*_*ST*_ values were arranged in descending order. The first 5% was regarded as the significant window (Figure 3A), and a total of 1148 genes were obtained. Under the 1% threshold, 184 candidate genes were screened out with iHS (Figure 3B). A total of 29 genes were obtained from the intersection of the two gene sets (Figure 3C), scuh as *BMPR1B*, *3BHSD*, *STPG2*, *ATRN*, *GANS*, etc (*Tabel A2. xlsx*).

### 3.5 Functional annotation of genes

**FIGURE 4:**
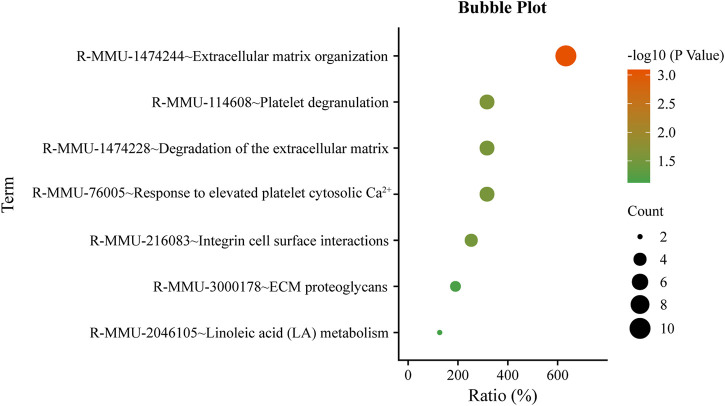
The Reactome pathways of 184 candidate genes screened out with iHS.

**FIGURE 5:**
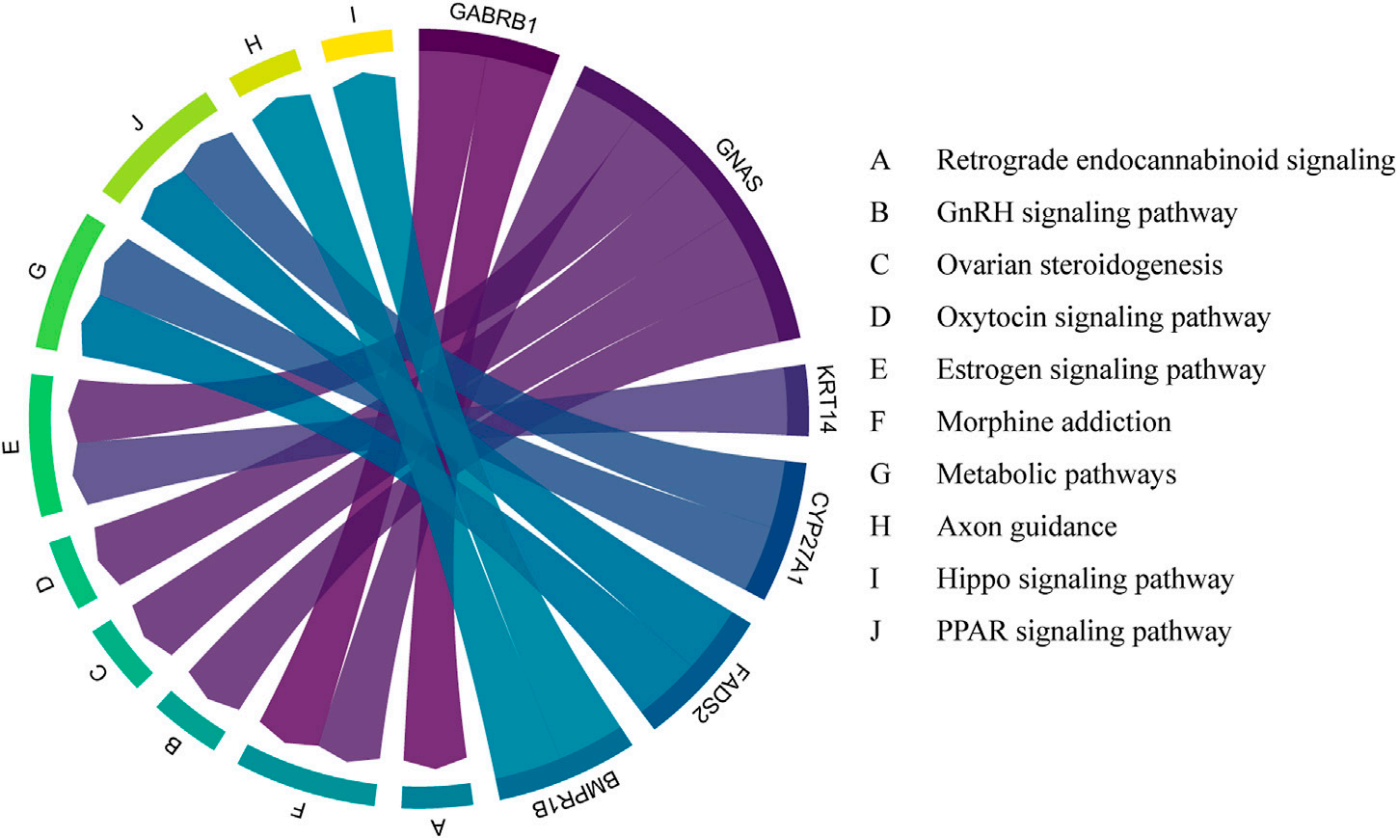
Enrichment pathways of 29 genes obtained by intersection of *F*_*ST*_ and IHS and their relationship.

A total of 184 genes were screened by iHS and GO and Reactome pathway analysis were performed on the 184 genes. The enrichment results of the Reactome pathway of the candidate genes were shown in Figure 4. Some genes were related to animal reproduction (Table 2). Genes *ABCA4*, *ARL4C*, and *PARP14* can affect the function of ion channels. Genes *SOX2*, *DAB1*, and *COL5A2* regulate cell differentiation. 29 genes were obtained by the intersection of the results of *F*_*ST*_ and IHS, and after pathway enrichment, these genes were found to be related to GnRH signalingpathway, Ovarian steroidogenesis and Estrogen signaling pathway (Figure 5), indicateing that these genes may control PRS prolific trait.

**TABLE 2:** Candidate genes related to reproduction.

Gene symbol	NCBI gene ID	Go term name	Go term ID	OAR	Coordinates (bp)
*PAX5*	101108719	developmental process involved in reproduction	GO:0003006	2	51663045–51849640
		reproductive process	GO:0022414		
		sexual reproduction	GO:0019953		
*HERC2*	101102534	developmental process involved in reproduction	GO:0003006	2	113570525–113814058
		reproductive process	GO:0022414		
		Reproduction	GO:0000003		
		multicellular organism reproduction	GO:0032504		
		multi-organism reproductive process	GO:0044703		
*HSPA5*	780447	reproductive structure development	GO:0048608	3	10783159–10787445
		developmental process involved in reproduction	GO:0003006		
		reproductive process	GO:0022414		
		multicellular organismal reproductive process	GO:0048609		
*INSR*	443431	reproductive structure development	GO:0048608	5	13788125–13936266
		developmental process involved in reproduction	GO:0003006		
*CAST*	443364	reproductive process	GO:0022414	5	93695915–93785491
		sexual reproduction	GO:0019953		
*BMPR1B*	443454	reproductive structure development	GO:0048608	6	30030664–30482585
		developmental process involved in reproduction	GO:0003006		
		reproductive process	GO:0022414		
		cellular process involved in reproduction in multicellular organism	GO:0022412		
		Reproduction	GO:0000003		
		reproductive system development	GO:0061458		
		multicellular organism reproduction	GO:0032504		
		multi-organism reproductive process	GO:0044703		
*GABRB1*	101122358	Reproduction	GO:0000003	6	66336302–66776044
		multicellular organism reproduction	GO:0032504		
*HAS2*	101110341	multicellular organism reproduction	GO:0032504	9	31108300–31140164
*TLR5*	554256	reproductive structure development	GO:0048608	12	25835725–25861758
		reproductive system development	GO:0061458		
*C11ORF74*	101107893	developmental process involved in reproduction	GO:0003006	15	65840095–65907755
		reproductive process	GO:0022414		
		Reproduction	GO:0000003		
		multi-organism reproductive process	GO:0044703		
*FGF10*	443074	reproductive structure development	GO:0048608	16	30604216–30700637
		developmental process involved in reproduction	GO:0003006		
		reproductive process	GO:0022414		
		reproduction	GO:0000003		
*ABHD2*	101103014	developmental process involved in reproduction	GO:0003006	18	19910266–20020987
		reproductive process	GO:0022414		
		cellular process involved in reproduction in multicellular organism	GO:0022412		
		Reproduction	GO:0000003		
		sexual reproduction	GO:0019953		
		multicellular organism reproduction	GO:0048609		
		multicellular organismal reproductive process	GO:0044703		
		multi-organism reproductive process			
*ZFP42*	101110027	reproductive structure development	GO:0048608	26	16740249–16747149
		developmental process involved in reproduction	GO:0003006		
		reproductive process	GO:0022414		

### 3.6 PCR product sequencing and genotyping

**FIGURE 6:**
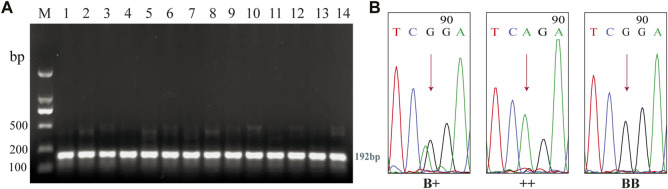
PCR amplification and sequencing of BMRPR1B. **(A)**, Detection of PCR amplification products of BMPR1B gene in PRS. M, DL2000DNA Marker. 1-14, PCR amplification product of *BMPR1B* gene. **(B)**, Sequencing results of the B+, ++, and BB genotypes.

After 1.5% agarose gel electrophoresis, the PCR products were found to conform to the expected size, indicating that the target fragment was successfully amplified (Figure 6A). The sequencing results are shown in Figure 6B. SNP locus detection revealed that BMPR1B gene G.431965A > G, and three genotypes, B+, ++, and BB, were detected (Table 3). The number of lambs of the BB genotype was significantly higher than that of the B+ and ++ genotypes (*p* < 0.05). The number of lambs of the B+ genotype was significantly higher than that of the ++ genotype (*p* < 0.05). It was shown in the *χ* ^2^ test that the PRS reached Hardy-Weinberg equilibrium at the *Fec* ^*B*^ locus.

**TABLE 3:** The genotype frequency and litter size of different BMPR1B genotypes of Pishan Red sheep.

Genotype	Number of samples	Genotype frequency	Allele frequency		Average number of lambs	*χ* ^2^
			B	+		
B+	64	0.492	0.5077	0.4923	1.453 ± 0.063^*a*^	1.1151
BB	34	0.262			1.735 ± 0.077^*b*^	
++	32	0.246			1.125 ± 0.059^*c*^	

Note: Different letters of shoulder labels of data in the same column indicate significant difference (*p* < 0.05).

## 4 Discussion

The Pishan Red sheep is a newly discovered local sheep group known for its stress resistance, perennial estrus, and high fecundity. Understanding the breed characteristics and evolutionary history of PRS will help to further strengthen the conservation and utilization of its genetic resources. PCA, N-J tree and Admixture suggest that PRS and QR could be subdivided into two genetic clusters, and have similar ancestral components.

### 4.1 LD and *Ne* population size

In PRS, LD was only moderate at 0–10 Kb and rapidly decreased to 0.120 ± 0.158 at 25–50 Kb. The *r* ^2^ we calculated for PRS was close to that of other sheep breeds and species. In Iranian Zandi sheep, the average *r* ^2^ for the pairwise space of 0–10 Kb was 0.26 (Ghoreishifar et al., 2019). In the Barbaresca sheep, the average *r* ^2^ for the intermarker distance of 0.5–1.0 Mb was 0.12 (Mastrangelo et al., 2017). However, in Border Leicester and Poll Dorset, the average *r* ^2^ for the intermarker distance of 0–10 Kb was 0.34 and 0.33, respectively (Al-Mamun et al., 2015). The average *r* ^2^ for the intermarker distances of 0–10 Kb and 10–25 Kb was 0.43 and 0.26, respectively, in Vrindavani cattle (Singh et al., 2021). In Gir cattle, the average *r* ^2^ value of 0.5–1 Mb was 0.032 (Ospina et al., 2019). Thus, the variation in the reported *r* ^2^ in different breeds and species suggests that LD are highly specific in sheep breeds. The PRS is located at the edge of the Taklimakan Desert. Its unique geographical environment may be the main reason for its high genetic diversity.

Mean *r* ^2^ values varied on chromosomes (from 0.182 ± 0.269 in OAR25 to 0.285 ± 0.367 in OAR19, distance <10 Kb), consistent with previous reports in sheep (Liu et al., 2017), beef cattle (Edea et al., 2014), and dairy cows (Qanbari et al., 2009). This phenomenon may be due to differences in recombination rates in different chromosomes, natural or artificial selection, and genetic drift (Mastrangelo et al., 2017). In the Vrindavani cattle, the highest mean *r* ^2^ value of chromosome 28 was 0.643, and the lowest *r* ^2^ value of chromosome 18 was 0.172 (0–10 Kb) (Singh et al., 2021). Moreover, the variation in *r* ^2^ estimated for different chromosomes was higher in short SNP pair distances, which is in line with the results reported for Chinese Merino sheep (Xinjiang type) (Liu et al., 2017). As seen from the attenuation of LD of 26 chromosomes of PRS, the LD of each chromosome was weak. The *r* ^2^ values were higher where the distance between the marked sites was close, but there were also higher *r* ^2^ values between the two sites, indicating a certain pattern of LD between the distant sites. The weak LD levels of OAR12, OAR18, OAR20, and OAR25 indicated that the degree of purification of these chromosomes was not high, leading to higher genetic diversity than other chromosomes. The chromosomes with higher *r* ^2^ values were OAR19, OAR15, OAR13, and OAR14, indicating that these four chromosomes may be more strongly selected than the other chromosomes.

The *Ne* of 236.99 of PRS 50 generations ago was similar to that of Chinese Merino sheep (Liu et al., 2017). We observed that *Ne* decreased more strongly from about 380 generations ago, consistent with the results of Chinese Merino sheep (Liu et al., 2017). The low level of *r* ^2^, even at relatively short distances, showed that the *Ne* in PRS was large in recent past generations compared with other species. For example, the *r* ^2^ for SNP pairs within 0.9–1.0 Mb and the *Ne* in recent generations in Duroc pigs were reported to be 0.2 and 75, respectively (Grossi et al., 2017). However, given the sharp drop in *Ne* in recent generations, we should be careful to maintain *Ne* larger than 100 individuals.

### 4.2 Adaptive mechanisms of the desert environment

The environmental adaptability differs between the Taklimakan Desert sheep breeds and those of other areas. PRS can adapt to extreme conditions, such as high salinity, drought, and ultraviolet rays. Under the iHS 1% threshold, 184 genes were screened. It was revealed that the genetic evidence and physiological mechanism of PRS adaptation to the desert environment. In terms of linoleic acid (LA) metabolism (R-MMU-2046105), *FADS1* and *FADS2* control polyunsaturated fatty acids (Hanson and Korotkova, 2002; Lawrence and Lawrence, 2004; Das, 2006; Calder, 2015), which accumulate fat to cope with extreme weather. The activation of phospholipase C enzymes results in the generation of second messengers of the phosphatidylinositol pathway in terms of adaptability. The events resulting from this pathway increase intracellular calcium and protein kinase C (PKC) activation. Phospholipase C cleaves the phosphodiester bond in PIP2 to form 1,2 diacylglycerol (DAG) and 1,4,5-inositol trisphosphate (IP3). IP3 opens *Ca* ^2+^ channels in the platelet-dense tubular system, raising intracellular *Ca* ^2+^ levels (R-MMU-76005). In terms of immunity, *HERC2* and *USP10* can promote the activation of the ATR-CHK1 pathway, triggering cell cycle checkpoints (Yuan et al., 2014). *PAALD* is involved in the process of phagocytosis (Sun et al., 2017). *KIF2A*, *TRIM66*, *BCL11B*, and *CLEC14A* play essential roles in repairing damaged cell DNA (Chen et al., 2019; Seira et al., 2019; Valisno et al., 2021; Su et al., 2021). *GPRIN3* and *HERC3* control cell senescence and apoptosis (Chen et al., 2018; Ding et al., 2021). In terms of growth and development, *ZFP42* can control the differentiation of embryonic stem cells (Scotland et al., 2009). *EVC* can promote chondrogenesis (Lamuedra et al., 2022). *GABRB1* correlates with hypothalamic volume and regulates intelligence (Zhu et al., 2014).

### 4.3 Genetic mechanisms of perennial estrus and reproduction

The estrus cycle refers to the time between the previous and next ovulation. The ovary undergoes follicular growth, maturation, ovulation, luteal formation, and degeneration during the estrus cycle. The vast majority of sheep are singleton and seasonally estrus, leading to the failure of a balanced supply of lamb meat in the four seasons, seriously restricting the production efficiency of the meat sheep industry. PRS live in desert environments, and after natural and artificial selection, it forms the characteristics of perennial estrus and the early onset of puberty. *TLR5* can effectively alleviate the stimulation caused by radiation (Brackett et al., 2021), and *SOX10* independently regulates the expression of *IRF1* in melanoma through the JAK-STAT signaling pathway (Yokoyama et al., 2021). *ATP6V0A* can affect fetal brain development (Aoto et al., 2021). *AUH* can cause the early onset of puberty (Bizjak et al., 2020). During the luteal phase of the estrus cycle, IFNE is highly expressed in the uterus and has a protective effect against uterine infection (Fischer et al., 2018). As a member of the BMP system, *BMPR1B* plays a significant role in the sheep ovary. The BMP system can control the proliferation and differentiation of ovarian granulosa cells and the development of oocytes, among which *BMPR1B* plays an essential role in the regulation of ovarian function. The SNP of *BMPR1B* C.746 A > G, the 249th amino acid change, partially inactivates *BMPR1B* protein. This change affects the reaction of the ligands *GDF5* and *BMP4* recognized by *BMPR1B* to steroid production, making follicles mature earlier and increasing the ovine ovulation number (Kumar et al., 2020). *BMPR1B* is the primary gene affecting the trait of lambing abundance in sheep (Mulsant et al., 2001; Al-Samerria et al., 2015).

Fibroblast growth factor (FGF) is a large family of paracrine cells that can regulate follicular development and oocyte maturation. *FGF10* can interact with *BMP15* to increase cumulus cell diffusion and improve glucose utilization (Caixeta et al., 2013). *FGF10* was expressed in oocytes and membrane cells in cattle follicles, acting on granulosa cells to inhibit steroid production. *FGF10* regulates the cumulus-oocyte complex, improving expansion and development ability (Zhang et al., 2010; Zhang et al., 2020). *FGF10* can also reduce the proportion of apoptotic oocytes and increase the number of cells developing to the blastocyst stage (Pinto et al., 2014). In embryonic development, *FGF10* can activate the MAPK pathway, increase the phosphorylation level of MAPK, and mediate the migration of sheep trophoblast cells (Yang et al., 2011). *FGF10* can reduce the level of FSHR mRNA in granulosa cells *in vitro*. *FGF10* can also inhibit the secretion of estrogen. The injection of *FGF10* at the initial stage of follicular selection can inhibit follicular growth, which may be achieved by reducing FSHR mRNA levels and thus inhibiting estrogen expression. At the same time, the *FGF10* mRNA concentration in the ovaries of healthy and growing bovine follicles was higher than that in atresia follicles (Buratini et al., 2007), which indicated that *FGF10* played an inhibitory role in early follicular development and promoted follicular maturation in late follicular development. *FGF10* can also regulate the expression of genes *CD9*, *CD81*, *DNMT1*, and *DNMT3B* to improve embryo quality (Pan et al., 2019).

Hyaluronic acid (HA), also called hyaluronate or hyaluronan, is an essential extracellular matrix component widely available in various mammalian tissues (Rodgers and Irving-Rodgers, 2010). HA is synthesized by three HA synthase families (*HAS1*, *HAS2*, and *HAS3*). *HAS2* is so critical to development that the HAS2-HA system is considered an essential HAS-HA system. *HAS2* is responsible for the rapid hyaluronic acid synthesis in cumulus-oocyte complexes and granulosa cells. In the dominant follicles of mammals, most of the hyaluronic acid is secreted by cumulus cells and is also present in the follicular fluid. *HAS2* plays a vital physiological role in oocyte maturation, ovulation, *in vivo* fertilization, and early embryonic development (Irving-Rodgers and Rodgers, 2006). *C11orf74* enhances sperm motility and improves fertilization (Majkowski et al., 2018). Progesterone can upregulate *ABHD2* to activate the camp-PKA signaling pathway (Jiang et al., 2021). These genes were strongly selected, suggesting that perennial estrus may result from a combination of these genes.

The number of lambs is a complex character affected by many factors, among which heredity is the main factor. The *Fec* ^*B*^ gene is the most studied gene and can significantly affect the ovulation number and lambing number of sheep breeds. The B+ genotype exists among multiple sheep breeds in Xinjiang, China, including Duolang and Hotan sheep. The number of lambs of the B+ genotype was significantly higher than that of the ++ genotype. We detected *Fec* ^*B*^ in 130 leather-red sheep and found three genotypes: BB, B+, and ++. The average number of lambs born in the BB genotype was the highest, followed by the B+ and ++ genotypes, indicating that the *Fec* ^*B*^ gene is an effective gene for improving the fecality of PRS.

## 5 Conclusion

In the extreme environment of the desert, PRS has formed the characteristics of perennial estrus, multiple pregnancies, and adequate resistance to stress. The LD decay rate of the PRS was faster, and LD and the *Ne* were at a relatively low level, suggesting that we should take reasonable protection measures to increase the PRS population. Genomic selection signal analysis and population validation showed that *Fec* ^*B*^ could be used as a molecular breeding marker for multiple fetal lines of PRS in desert environments.

## Data Availability

The datasets presented in this study can be found in online repositories. The names of the repository/repositories and accession number(s) can be found below: Our data has been uploaded to this website https://figshare.com/articles/dataset/e_vcf/21700937.
