# Supplementary material for: Population structure, genetic diversity and prolificacy in pishan red sheep under an extreme desert environment
Source: Front Genet. 2023 Apr 11;14:1092066. doi: 10.3389/fgene.2023.1092066 (PMC10126422; doi:10.3389/fgene.2023.1092066)
Supplement: Supplementary file 1 [file DataSheet1.PDF]

Supplementary Material

1 SUPPLEMENTARY TABLES 1

This section includes BMPR1B primer information and gene selective sweep results.

Table S1. Primer of BMPR1B gene.

Gene	Primer sequence (5'→3')	Fragment size/bp	Annealing temperature °C
BMPR1B	TGGAAAAGGTCGCTATGGGG<br>AGCTAGGAAACCCTGAACATCG	192	60

2 SUPPLEMENTARY TABLES 2

Tabel A2.xlsx: Gene selective sweep results.
